# Human immune responses to *Plasmodium falciparum* infection: molecular evidence for a suboptimal THαβ and TH17 bias over ideal and effective traditional TH1 immune response

**DOI:** 10.1186/1475-2875-12-392

**Published:** 2013-11-05

**Authors:** Wan-Chung Hu

**Affiliations:** 1 Department of International Health, Johns Hopkins University School of Public Health, Baltimore, MD 21205, USA; 2 Department of Neurology, Shin Kong Memorial Hospital, No.95, Wen Chang Road, Shih Lin District, Taipei, Taiwan

**Keywords:** TH1, TH2, TH17, THαβ, Malaria, Immune response

## Abstract

**Background:**

Using microarray analysis, this study showed up-regulation of toll-like receptors 1, 2, 4, 7, 8, NF-κB, TNF, p38-MAPK, and MHC molecules in human peripheral blood mononuclear cells following infection with *Plasmodium falciparum*.

**Methods:**

This analysis reports herein further studies based on time-course microarray analysis with focus on malaria-induced host immune response.

**Results:**

The results show that in early malaria, selected immune response-related genes were up-regulated including α β and γ interferon-related genes, as well as genes of IL-15, CD36, chemokines (CXCL10, CCL2, S100A8/9, CXCL9, and CXCL11), TRAIL and IgG Fc receptors. During acute febrile malaria, up-regulated genes included α β and γ interferon-related genes, IL-8, IL-1b IL-10 downstream genes, TGFB1, oncostatin-M, chemokines, IgG Fc receptors, ADCC signalling, complement-related genes, granzymes, NK cell killer/inhibitory receptors and Fas antigen. During recovery, genes for NK receptorsand granzymes/perforin were up-regulated. When viewed in terms of immune response type, malaria infection appeared to induce a mixed TH1 response, in which α and β interferon-driven responses appear to predominate over the more classic IL-12 driven pathway. In addition, TH17 pathway also appears to play a significant role in the immune response to *P. falciparum*. Gene markers of TH17 (neutrophil-related genes, TGFB1 and IL-6 family (oncostatin-M)) and THαβ (IFN-γ and NK cytotoxicity and ADCC gene) immune response were up-regulated. Initiation of THαβ immune response was associated with an IFN-αβ response, which ultimately resulted in moderate-mild IFN-γ achieved via a pathway different from the more classic IL-12 TH1 pattern.

**Conclusions:**

Based on these observations, this study speculates that in *P. falciparum* infection, THαβ/TH17 immune response may predominate over ideal TH1 response.

## Background

TH1 has been suggested to be the dominant and protective immune response against malaria both in rodents and humans [1,2]. Yet the blood stage of *Plasmodium falciparum* can serve to immunosuppress the host’s immune response to the liver stage of the parasite [3] Dendritic cell maturation is inhibited by *P. falciparum*-infected red blood cells (RBCs) [4]; monocyte maturation is also suppressed by malaria pigments (haemozoin), and low serum IL-12 was reported in severe malaria [5,6]. These observations suggest that the IL-12-driven TH1, IFNγ-dominant response patterns seen following *P. falciparum* infection may not be functioning optimally; thus, against this background data, one may ask how the infected host is capable of mounting an effective TH1 response against the blood-borne stage of this parasite? An alternative pathway may be needed to achieve this host immune response.

To gain a better understanding of host immune response patterns associated with *P. falciparum* infection in humans, this study carried out transcriptional profiling using microarray analysis of peripheral blood mononuclear cells (PBMCs) after *P. falciparum* infection. The earlier analysis showed an up-regulation of gene expression for toll-like receptor signalling, NF-κB, TNF, IFN-γ, IL-1β, p38 MAPK, MHC class I and II molecules [7]. This study further analysed the data focusing on the specific type or types of host immune response induced by infection. Results indicated an up-regulation of TGF-β and the IL-6 family gene (oncostatin M), both of which induce TH17 immune response. This study also observed an elevation in the expression of interferon gamma, NK cell cytotoxicity, antibody-dependent cell cytotoxicity (ADCC), which are triggered by interferon alpha/beta (THαβ), instead of IL-12 (TH1). These results suggest multiple immune pathways may be activated following infection with *P. falciparum*. For the purpose of this study have classified these additional pathways as: THαβ and TH17 immune response. The THαβ pathway is not generally recognized within the immunology community as a distinct T cell maturation pathway while the TH17 response pattern has only recently been recognized [8]. However, THαβ responses in which α and β interferons drive a succession of cellular process leading to mid-moderate IFN-γ production and NK cell and ADCC activation have been described in the host response to viruses but they have not been shown to play an important role in the maturation of the anti-malaria adaptive immune response.

To further clarify this point, traditionally TH1 leads to immune response against intracellular bacteria and protozoa, and THαβ against viral infections. Macrophage activation is characteristic of traditional TH1 immune response, while NK activation (natural cytotoxicity and ADCC) is more characteristic of THαβ immune response. In addition, TH17 immune response is typical immunological pathway against extracellular bacteria and fungi. Pathogens may induce ineffective host immune response for immune evasion. Thus, malaria infection appears to be somewhat unique in that it appears to elicit a combined response reflective of both TH17 and THαβ host immune response alternative to effective TH1 immune response.

## Methods

### Ethical statement

This study was approved by Johns Hopkins University School of Public Health Institutional Research Board and Cameroon Department of Health Institutional Research Board. Written informed consent was obtained from each individual of this study.

### Previous analysis

Subject recruitment, sample collection and preparation, and RNA [ribonucleic acid] purification were described previously in Ockenhouse et al. [7]. Briefly, two groups of subjects were recruited for this study after Johns Hopkins University Institutional Review Board and US Army HSRRB ethical approvals were obtained. In the study cohort, 22 subjects, 20–45 years of age, were recruited from the Walter Reed Army Institute of Research (WRAIR). Subjects agreed to receive mosquito bites from laboratory-reared *Anopheles stephensi* infected by *P. falciparum* (3D7 strain). Once parasitaemia was detected in the subjects’ peripheral blood, the subjects received treatment with chloroquine. Blood samples were drawn during the un-infected baseline period (U) and again, when parasitaemia was found for the first time (early malaria (E)).

In the other study cohort, 15 adults were recruited from Cameroon. These subjects were 19–49 years of age with acute *P. falciparum* infection. All suffered from typical relapsing fever and blood smears showed parasitaemia. Patients were excluded if they actively suffered from other infectious or inflammation diseases. These subjects received at least one week of anti-malarial drug treatment (Cotecxin). Blood drawing was performed during the acute febrile infection period (A) and one month later during the recovery period (R). During the recovery period, physical examinations and blood smears were performed to ensure that malaria symptoms were no longer present and parasitaemia was no longer detectable.

In the first study cohort, PBMCs were separated from whole blood samples by Ficoll-gradient at WRAIR. In the second study cohort, blood was collected in CPT [Cell Preparation Tube] tubes and PBMCs were isolated after centrifuge. A RNA stabilizing reagent-RNA later (Ambion, CA, USA) was added and samples were shipped to the USA on dry ice. Total RNA was extracted from both sets of samples using Trizol. The quality of sample RNA was estimated by spectrometry (OD > 1.8) and gel electrophoresis.

### Microarray preparation

Affymetrix U133A GeneChips (Affymetrix, Santa Clara, CA, USA) were used in this study. The GeneChips contain 22,283 probe-sets, including 14,500 known well-characterized human genes and 18,400 transcripts. Before chip hybridization, a QIAGEN RNeasy clean-up kit was used to purify total RNA. Processing of templates for analysis on the Affymetrix U133A GeneChip was performed in accordance with methods described in the Affymetrix Technical Manual, Revision Three. Total RNA from the blood samples were hybridized into the arrays. Detailed cDNA preparation, *in vitro* transcription, staining, and scanning of Affymetrix U133A GeneChips were described previously in Ockenhouse et al. [7] paper.

### Data analysis

This study used GeneSpring software and GeneSpring default normalization to perform one-way ANOVA tests, using un-infected samples as baseline. Significantly up-regulated genes were selected if false discovery rate (FDR) was < 0.05 and fold change was >1.5 × when compared to un-infected baseline. These genes were placed into three groups based on expression levels in different stages of the infection. In addition, Pearson’s correlation analysis and Rank correlation analysis were performed to find the relationship between expression levels of genes after malaria infection. Pathway analysis was performed by using the Pathway Architect (Stratagene Inc,CA, USA) software to identify the specific immunological pathways involved.

### Microarray validation

In order to validate the study’s microarray results, several assays were used including flow cytometry and commercial kits for plasma protein expression. Peripheral blood mononuclear cells analysis was done by a FACScan flow cytometer (Becton-Dickinson, Mountain View, CA) using a panel of mouse anti-human monoclonal antibodies such as ICAM1 antibody before and after malaria challenge. Measuring the chemokine levels including CXCL10, CXCL9, and CCL2 in paired plasma samples collected before and after malaria challenge were done by using commercial kits (R&D systems, Minneapolis, MN). These validation assays were described in the previous Ockenhouse et al. [7] paper.

### Microarray accession numbers

The Affymetrix data sets can be accessed at Gene Expression Omnibus [http://www.ncbi.nlm.nih.gov/geo/] under the accession number GSE5418.

## Results

A total of 2,894 genes were differentially expressed out of the 22,283 probe sets in Affymetrix U133A GeneChip based on the criteria of data analysis. Gene expression patterns were measured in individuals with pre-symptomatic, experimental, early malarial infection and in subjects with naturally acquired acute febrile malarial infection. A baseline set of data derived from 22 uninfected healthy US subjects was used for gene comparison in samples from early malarial infection, acute febrile malarial infection and recovery stage. This baseline dataset is considered appropriate for comparisons between Cameroonian samples and USA samples because of the difficult in collecting baseline samples from Cameroonian subjects that have had no prior exposure to malaria. As indicated previously, the study site in Cameroon is endemic for malaria so it is difficult to find adults in the area that may not already be in the prodromal phase of malaria. The correlation of the average gene expression of baseline samples to the average gene expression from 22 USA subjects and from 15 Cameroonian adults exceeded 97%, indicating comparability of gene expression between datasets collected and processed at the WRAIR for US volunteers and at the Johns Hopkins Bloomberg School of Public Health for Cameroonian subjects.

### TH1 immune response-related gene up-regulation during malaria

**Figure 1:**
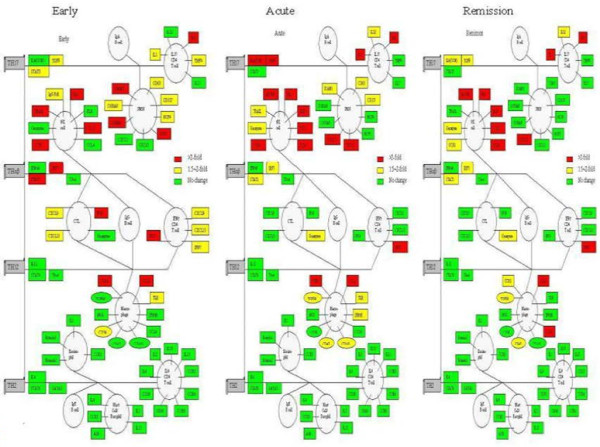
**Summary of gene expression of four immunities of malaria.** Summary of gene expression of the four immunological pathways during the early malaria, acute febrile malaria, and recovery period compared to un-infection baseline. Red means gene expression with greater than two-fold up-regulation. Yellow means gene expression with 1.5-2-fold up-regulation. Green means gene expression was not changed. Square shape means genes which activate the cell, and ellipse shape means genes which suppress the cell. Although interferon alpha/beta was not detected, (IRF7) means that this up-regulated gene regulates the transcription of interferon alpha/beta.

After *P. falciparum* infection, many immune response-related genes of the TH1 immunological pathway were up-regulated (Table 1). Although interferon alpha/beta was not detected in this study, the major transcription factor of interferon alpha/beta synthesis, IRF7, was up-regulated after malaria. Many interferon alpha/beta inducible genes were up-regulated during the early stage of malaria including: tryptophenyl-tRNA (IFI53), interferon alpha inducible protein 27 (IFI27), interferon-induced protein 44 (IFI44), interferon-stimulated protein 15KD (G1P2), interferon regulatory factor 1 (IRF1), 2′-5′-oligoadenylate synthetase 3 (OAS3), signal transducer and activator of transcription 1 (STAT1), interferon stimulated protein 35 (IFI35), Myxovirus resistance 1 (MX1), interferon-induced protein with tetratricopeptide repeat 4 (IFIT4), interferon-induced protein with tetratricopeptide repeat 1 (IFIT1), and interferon-induced protein with tetratricopeptide repeat 2 (IFIT2). The expression levels of the above genes were greater than two-fold in early malaria, as compared to un-infection baseline. In acute febrile malaria, the expression levels of most of the alpha/beta interferon genes tended to decline and all returned to baseline levels of expression during the recovery period (Table 1). Summary of all four immunological pathways in malarial infection is shown in Figure 1.

**Table 1:** TH1 (primarily THαβ) immune response related gene up-regulation after malaria

**U**	**E**	**E/U**	**A**	**A/U**	**R**	**R/U**	**Gene**	**TH1 Immune response**
0.595	3.656	**6.144**	1.213	**2.038**	1.003	*1.685*	IFI53	IFNa/b
0.898	3.089	**3.439**	1.298	1.445	1.132	1.260	IFI27	
0.825	2.375	**2.878**	1.077	1.305	0.928	1.124	IFI44	
0.552	2.093	**3.791**	1.158	**2.097**	0.933	*1.690*	G1P2	
0.51	2.07	**4.058**	1.031	**2.021**	0.947	*1.856*	IRF1	
0.813	1.763	**2.168**	1.08	1.328	0.926	1.138	OAS3	
0.579	1.829	**3.158**	1.058	*1.827*	0.977	*1.687*	STAT1	
0.722	1.773	**2.455**	1.091	*1.511*	0.952	1.318	IFI35	
0.881	1.586	*1.800*	1.069	1.213	0.898	1.019	OASL	
0.672	1.498	**2.229**	1.046	*1.556*	0.954	1.419	MX1	
0.662	1.532	**2.314**	1.26	*1.903*	1.097	*1.657*	IRF7	
0.898	4.549	**5.065**	0.992	1.104	0.894	0.995	IFIT4	
0.902	2.352	**2.607**	0.938	1.039	0.924	1.024	IFIT1	
0.931	1.935	**2.078**	1.065	1.143	0.964	1.035	IFIT2	
0.629	1.214	*1.930*	1.149	*1.826*	1.019	*1.620*	IFITM1	
0.655	1.043	*1.592*	2.424	**3.700**	1.694	**2.586**	IFNG	IFNg
0.587	6.22	**10.596**	1.099	*1.872*	0.823	1.402	GBP1	
0.527	1.908	**3.620**	1.111	**2.108**	0.947	*1.796*	GBP2	
0.755	1.273	*1.686*	1.182	*1.565*	0.951	1.259	IFNGR2	
0.658	1.31	*1.990*	1.284	*1.951*	0.916	1.392	IFI30	
0.781	1.333	*1.706*	1.322	*1.692*	1.123	1.437	JAK2	
0.72	2.049	**2.845**	0.991	1.376	0.95	1.319	TAP1	
0.781	1.371	*1.755*	1.057	1.353	1.067	1.366	JAK1	
0.838	1.261	*1.504*	1.106	1.319	0.9	1.073	FcGR2B	ADCC
0.863	1.199	1.389	1.925	**2.230**	1.075	1.245	FcAR	
0.514	0.911	*1.772*	1.658	**3.225**	1.339	**2.605**	FcGR3B	
0.708	1.208	*1.706*	1.132	*1.598*	0.896	1.265	FcGR2A	
0.64	4.954	**7.740**	1.265	*1.976*	0.849	1.326	FcGR1A	
0.751	1.285	*1.711*	1.412	*1.880*	1.035	1.378	C1QA	
0.765	1.601	**2.092**	1.073	1.402	0.946	1.236	FcGR3A	
0.597	1.237	**2.072**	1.658	**2.777**	1.339	**2.242**	FcER1G	
0.953	0.658	0.690	1.298	1.362	1.502	*1.576*	CD3z	
0.819	0.676	0.825	1.455	*1.776*	1.329	*1.622*	Fyn	
0.656	1.029	*1.568*	1.219	*1.858*	1.035	*1.577*	DAP12	
0.848	0.985	1.161	1.271	*1.498*	1.204	1.419	Syk	
0.566	0.449	0.793	2.044	**3.611**	1.829	**3.231**	PI3K	
0.748	1.108	1.481	1.152	*1.540*	1.03	1.377	Rac1	
0.83	1.21	1.457	1.25	*1.506*	1.13	1.361	PAK1	
0.823	0.807	0.980	1.464	*1.778*	1.191	1.447	MAP2K1	
0.721	0.872	1.209	1.516	**2.102**	1.375	*1.907*	MAP2K2	
0.644	0.81	1.257	1.547	**2.402**	1.382	**2.145**	Arf6	
0.881	0.642	0.728	2.723	**3.090**	2.931	**3.326**	KLRC3	NK cell
0.829	0.868	1.047	1.26	*1.519*	1.492	*1.799*	NKEFB	
0.927	0.848	0.914	1.442	*1.555*	1.432	*1.544*	KIR2DL3	
0.857	0.694	0.809	2.199	2.565	2.1	2.450	KIR3DL2	
0.77	3.235	**4.201**	0.835	1.084	0.798	1.036	TNFSF10	
0.653	1.242	*1.901*	1.152	*1.764*	1	*1.531*	Fas	
0.693	0.991	1.430	1.144	*1.650*	1.084	*1.564*	MICB	
0.901	0.772	0.856	1.439	*1.597*	1.31	1.453	ETS1	
0.704	0.899	1.276	1.596	**2.267**	1.379	*1.958*	CEBPg	
0.757	1.145	*1.512*	1.119	1.478	1.02	1.347	MEF	
0.638	0.765	1.199	1.674	**2.623**	1.458	**2.285**	ID2	
0.71	1.397	*1.967*	1.092	*1.538*	0.884	1.245	IL15	
0.908	0.738	0.812	1.521	*1.675*	1.626	*1.790*	GZMM	
0.768	0.962	1.252	1.568	**2.041**	1.199	*1.561*	CD58	
0.548	1.223	**2.231**	1.491	**2.720**	0.963	*1.757*	CCR1	TH1 Chemokine
0.381	0.974	**2.556**	2.085	**5.472**	1.713	**4.496**	CCL3	
0.689	0.735	1.066	3.206	**4.653**	2.65	**3.846**	CCL4	
0.986	5.194	**5.267**	1.045	1.059	0.956	0.969	CXCL10	
1.029	1.576	*1.531*	0.835	0.811	0.83	0.806	CXCL9	
0.824	1.265	*1.535*	0.924	1.121	0.808	0.980	CXCL11	
0.863	0.641	0.742	1.495	*1.732*	1.557	*1.804*	GZMK	CD8 T cell
0.795	0.651	0.818	1.98	**2.490**	1.739	**2.187**	GZMB	
0.881	0.638	0.724	1.236	1.402	1.522	*1.727*	GZMA	
0.829	0.765	0.922	1.432	*1.727*	1.385	*1.670*	RUNX3	
0.656	1.07	*1.631*	1.308	*1.993*	1.106	*1.685*	Rel	
0.925	0.812	0.877	1.497	*1.618*	1.593	*1.722*	CD3g	
0.871	0.892	1.024	2.021	**2.320**	1.719	*1.973*	CDC42	
0.749	0.692	0.923	1.885	**2.516**	1.524	**2.034**	Kras	
0.78	1.159	1.485	1.209	*1.55*	1.094	1.402	ICOS	
0.784	0.915	1.167	1.497	*1.909*	1.486	*1.895*	CD28	
0.77	1.007	1.307	1.224	*1.589*	1.038	1.348	Bcl10	
0.816	0.87	1.066	1.557	*1.908*	1.45	*1.776*	MALT1	

Bold means greater than 2-fold change.

Italic means 1.5-2-fold change.

Plane means no change.

Interferon gamma and many interferon gamma-related genes were also up-regulated after malaria. These observations are consistent with a model in which malaria infection induces primarily a TH1 type immune response. These up-regulated genes included: interferon gamma, interferon inducible guanylate binding protein 1 (GBP1), interferon inducible guanylate binding protein 2 (GBP2), Janus kinase1 (JAK1), and transporter with antigen processing 1 (TAP1). The expression levels of GBP1, GBP2, and TAP1 ranged from 10.6- to 2.8-fold above baseline during early stage of malaria, and the expression level of GBP2 was greater than two-fold change during acute febrile malaria. These interferon gamma inducible genes all returned to baseline levels of expression during the recovery periods of the disease. The up-regulation of a number of TH1 chemokines over the malaria disease course was also consistent with a TH1 model of anti-malaria immune response. Up-regulated TH1 related chemokine genes included: CXCL10, CCL3, CCL3 and CCR1. The expression levels of CXCL10 (5.3-fold) was highest during the early phase of malaria, while CCR3 and CCL4 were higher during febrile and recovery stages of the disease (Table 1).

There were many ADCC and NK cell-related genes up-regulated after malaria. ADCC-related genes included: Fc receptors (Fc alpha receptor, Fc gamma receptor 3B, Fc gamma receptor 1A, and Fc gamma receptor 3A), and ADCC signalling genes (CD3z, Fyn, DAP12, Syk, PI3K, Rac1, PAK1, MAP2K1, MAP2K2, and Arf6). NK cell-related genes included NK cell receptor-related genes (KLRC3and KIR3DL2), TNF superfamily ligand 10 (TRAIL), NK cell-related transcription factors (CEBPgamma,), interleukin 15, and NK cell-restricted granzyme M. Interleukin 15 can enhance NK cell differentiation and proliferation. ADCC is mainly mediated by NK cells, but it can also be mediated by macrophages. Fc receptor and ADCC signalling gene up-regulation is consistent with the ADCC machinery being turned on after *P. falciparum* infection. Among the above genes, gene expression levels were greater than two-fold change (range two- to seven-fold; see Table 1) in early malaria including: Fc gamma receptor 1A, Fc gamma receptor 3A, Fc epsilon receptor 1G, and TRAIL. Gene expression levels were greater than two-fold change in acute febrile malaria including: Fc alpha receptor, Fc gamma receptor 3B, Fc epsilon receptor 1G, PI3K, MAP2K2, Arf6, KLRC3, KIR3DL2, and CEBP gamma (range two- to four-fold). Gene expression levels were greater than two-fold change in the recovery period, including: Fc gamma receptor 3B, Fc epsilon receptor 1G, PI3K, Arf6, KLRC3, and KIR3DL2 range two- to three-fold). Although ADCC signalling genes were not greater than two-fold, they were higher than 1.5-fold change after malarial infection. CTL-related genes (CD8 T cells in Table 1) were only moderately up-regulated during malaria. Among these genes, granzyme B, CDC42, and Kras exhibited ≥ two-fold change during acute febrile malaria. Granzyme B and Kras were ≥ two-fold change in the recovery period.

Although macrophage/monocyte activation is traditionally considered to be a strong marker for classic TH1 type immune response, this study surprisingly found that express levels of most macrophage activation genes were relatively unchanged or slightly suppressed after *P. falciparum* infection (Table 2). These data support the hypothesis that there may be two pathways involved in the induction of TH1 types of immune responses: a THαβ interferon driven path and IL-12 driven path (see Discussion). Most macrophage activation and proliferation-related genes were unchanged (Table 2). IL-12 is clearly the major cytokine needed to initiate a strong classic TH1 type immune response, and IL-12 expression levels remain unchanged over the course of this study (Table 2). This observation suggests that the induction of a TH1-type anti-malarial immune response may be driven by cytokines other than IL-12. Like IL-12, other traditional TH1 cytokine: IL-3, M-CSF, and GM-CSF can also cause macrophage proliferation but these genes are also not up-regulated in response to infection with *P. falciparum* (Table 2). In contrast, many macrophage inhibition-related genes were up-regulated after malaria, including haem oxygenase 1 (HMOX1), JunB, and IRF1. JunB, PU.1 and IRF1 suppress macrophage proliferation. As indicated above, a macrophage differentiation inducer, MafB, is up-regulated after malaria. Its antagonists, JunB and PU.1, were also up-regulated, so the effect of MafB could be suppressed. PU.1 up-regulation can drive monocytes to differentiate into dendritic cells instead of macrophages. Although macrophages can also express Fc receptor genes, this study thinks these Fc receptor expression after malaria should mainly occur in NK cells. The Fc receptor signalling pathway (ADCC signalling pathway) in NK cell was all up-regulated (Table 1). Also, DAP12 up-regulation can suppress Fc receptor signalling in macrophages, so it is less likely that these up-regulated Fc receptors belong to macrophages. Although IL-10 itself was not up-regulated, many IL-10 downstream genes were up-regulated as shown in Figure 1 of the previous analysis results [7]. IL-10 can activate NK cells and de-activate macrophages. HMOX1 is the major downstream effector molecule to mediate IL-10 inhibition of macrophages. HMOX1 was up-regulated after malaria. HMOX1 generates CO molecule to mediate anti-inflammatory effects in macrophages, which is opposite to nitric oxide synthetase (iNOS), which generates NO molecule to cause inflammatory effects. Argininesuccinate synthetase (ASS) is the rate-limiting enzyme to synthesize arginine, the substrate of iNOS. iNOS is the central mediator representing macrophage classical activation, and unchanged ASS and iNOS express levels support the notion that macrophages are not being activated after malaria infection. In the study, macrophages were inhibited rather than activated after malaria. In summary, many elements of TH1 immune response were up-regulated following infection with *P. falciparum*, including: interferon alpha/beta inducible genes, interferon gamma inducible genes, NK cell-related genes (NK cell cytotoxicity), ADCC-related genes, TH1 chemokines, and CD8 T cell-related genes. However, macrophages appear to become de-activated and do not appear to proliferate after malarial infection.

**Table 2:** TH1 (primary TH12) immune response unchanged after malaria

0.755	1.273	*1.686*	1.182	*1.565*	0.951	1.259	IFNGR2	Macrophage
1.02	0.91	0.892	1.16	1.137	1.13	1.107	CD40L	Activation
0.804	1.271	*1.580*	1.132	1.407	1.098	1.365	TNFalpha	
0.97	1.04	1.072	1.01	1.041	1.03	1.061	TNFbeta	
0.733	1.263	*1.723*	1.293	*1.763*	0.864	1.178	TLR2	
0.744	1.125	*1.512*	1.385	*1.861*	0.991	1.331	TLR4	
0.739	1.051	1.422	1.408	*1.905*	1.044	1.412	TLR8	
0.99	1.04	1.050	1.03	1.040	1	1.010	IL12A	
1.02	1.04	1.019	0.96	0.941	0.95	0.931	IL12B	
1.13	1.08	0.955	0.96	0.849	0.94	0.831	IL12RB1	
1.04	1.16	1.115	1.02	0.980	0.97	0.932	IL12RB2	
1.04	0.78	0.75	1.22	1.173	1.27	1.221	STAT4	
1.03	1.08	1.048	1.02	0.990	1.01	0.980	iNOS	
1.054	1	0.948	1	0.948	1.04	0.986	ASS	
1	1.27	1.27	0.94	0.94	0.96	0.96	MMP9	
0.87	0.98	1.126	1.19	1.367	1.12	1.287	TRAF6	
0.98	1.03	1.051	0.99	1.010	1	1.020	TRANCE	
1.05	1.06	1.009	0.94	0.895	0.94	0.895	RANK	
0.99	1.039	1.049	0.97	0.979	0.99	1	ERM	
1.07	1.08	1.009	0.93	0.869	0.92	0.859	M-CSF	Macrophage
1.02	1.03	1.009	0.97	0.950	0.97	0.950	GM-CSF	Proliferation
1.11	1.03	0.927	0.94	0.846	0.89	0.801	IL-3	
0.92	1.04	1.130	1.13	1.228	1.04	1.130	RUNX1	
0.86	0.89	1.034	0.85	0.988	0.84	0.976	Myb	
1.21	1.25	1.033	0.81	0.669	0.87	0.719	Myc	
1	1.24	1.24	1	1	0.95	0.95	CEBPalpha	
1.04	1.04	1	1	0.961	1.03	0.990	HOXB7	
1.06	1.17	1.103	0.95	0.896	0.97	0.915	GATA1	
1.07	1.06	0.990	0.95	0.887	0.98	0.915	GATA2	
1.12	1.19	1.062	0.82	0.732	0.86	0.767	Tal1	
0.358	0.952	**2.659**	2.495	**6.969**	1.351	**3.773**	MafB	
0.841	0.809	0.961	1.401	*1.665*	1.295	*1.539*	TGFBR2	Macrophage
0.85	1.355	*1.594*	1.024	1.204	0.727	0.855	CD36	Inhibition
0.706	0.972	1.376	1.307	*1.851*	1.209	*1.712*	CD47	
0.943	0.783	0.830	1.521	*1.612*	1.097	1.163	CD163	
0.522	0.781	1.496	1.776	**3.402**	1.556	**2.980**	JunB	
0.51	2.07	**4.058**	1.031	**2.021**	0.947	*1.856*	IRF1	
0.73	0.95	1.301	2.62	**3.589**	1.81	**2.479**	HMOX1	
0.83	1.24	1.493	1.27	*1.530*	0.97	1.168	SIRPalpha	
0.7	1.319	*1.884*	1.48	**2.114**	1.02	1.457	PU.1	

Bold means greater than 2-fold change.

Italic means 1.5-2-fold change.

Plane means no change.

### TH17 immune response related gene up-regulation during malaria

A number of genes associated with the newly described TH17 immune response pathway were up-regulated after infection with *P. falciparum* (Table 3). Of these genes, IL-8 showed the greatest degree of up-regulation over the entire course of the disease cycle (early, febrile period and recovery periods), with expression levels peaking during the febrile period of illness (8.5-fold increase; Table 3). Other up-regulated genes included other TH17-related cytokines, IL1β, TGFβ1, and oncostatin M as well as TH17-related transcription factors, CEBP delta, and CEBP gamma. In addition, selected neutrophil-related genes were also up-regulated, including: neutrophil attracting chemokines, S100A9, CXCL2, and CXCL3, the neutrophil-related CD molecules, ICAM1, the NADPH oxidases gene NCF1, and acute reaction protein, PGE synthetase2. Many complement-related genes were also up-regulated including C3A receptor1, C5 receptor1, delay-accelerating factor (DAF), and Factor D.

**Table 3:** TH17 related gene up-regulation after malaria

**U**	**E**	**E/U**	**A**	**A/U**	**R**	**R/U**	**Gene**	**TH17Immune response**
0.767	0.775	1.010	1.275	*1.662*	1.382	*1.801*	IL32	IL17 CD4 Tcell
0.343	0.779	**2.271**	2.922	**8.518**	1.389	**4.049**	IL8	
0.805	1.265	*1.571*	2.276	**2.827**	1.931	**2.398**	IL1B	
0.804	1.271	*1.580*	1.132	1.407	1.098	1.365	TNFalpha	
0.653	1.019	*1.560*	1.346	**2.061**	1.156	*1.770*	TGFB1	
0.841	0.809	0.961	1.401	*1.665*	1.295	*1.539*	TGFBR2	
0.795	0.907	1.140	1.849	**2.325**	1.491	*1.875*	OSM	
0.77	1.333	*1.731*	1.04	1.350	0.865	1.123	STAT3	
0.764	0.894	1.170	2.98	**3.900**	1.648	**2.157**	CEBPD	
0.459	0.991	**2.159**	1.694	**3.690**	1.163	**2.533**	CEBPB	
0.903	1.552	*1.718*	0.899	0.995	0.787	0.871	S100A8	Neutrophil
0.707	1.568	**2.217**	1.049	1.483	0.852	1.205	S100A9	
0.667	0.97	1.454	2.827	**4.238**	1.699	**2.547**	CXCL2	
0.923	1.027	1.112	2.027	**2.196**	1.333	1.444	CXCL3	
0.745	1.4	*1.879*	1.182	*1.586*	0.946	1.269	CD63	
0.708	1.26	*1.779*	1.183	*1.670*	0.917	1.295	CD157	
0.722	1.948	**2.698**	1.155	*1.599*	0.923	1.278	ICAM1	
0.725	0.911	1.256	1.167	*1.609*	1.054	1.453	ITGB2	
0.954	2.945	**3.087**	0.939	0.984	0.801	0.839	NCF1	
0.827	1.471	*1.778*	1.116	1.349	0.953	1.152	NCF4	
0.742	1.047	1.411	1.202	*1.619*	1.011	1.362	CD97	
0.709	1.382	*1.949*	1.16	*1.636*	0.801	1.129	FPR1	
0.918	1.131	1.232	1.619	*1.763*	1.23	1.339	Pentraxin3	
0.723	1.08	1.493	2.135	**2.952**	1.895	**2.621**	PGES2	
0.635	1.087	*1.711*	2.079	**3.274**	1.218	*1.918*	C3AR1	Complement
0.623	0.908	1.457	2.628	**4.218**	1.571	**2.521**	C5R1	
0.552	0.881	*1.596*	2.297	**4.161**	1.392	**2.521**	DAF	
0.751	1.285	*1.711*	1.412	*1.880*	1.035	1.378	C1QA	
0.67	0.914	1.364	1.349	**2.013**	1.166	*1.740*	FactorD	
0.761	1.093	1.436	1.418	*1.863*	1.121	1.473	Properdin	

Bold means greater than 2-fold change.

Italic means 1.5-2-fold change.

Plane means no change.

### TH2 immune response related genes unchanged during malaria

Most TH2 immune response-related genes were remarkably unchanged over the course of the malaria disease cycle (Table 4). An extensive analysis of TH2 cytokines and chemokines, as well as Mast cell and eosinophil genes revealed that gene expression level were essentially unaffected by infection with *P. falciparum*, thus providing strong evidence that TH2-type immune response was not significantly initiated during malaria in this study. Expression levels for only a single gene involved in Mast cell activation, Fc epsilon receptor 1A surface receptor, was modified during the malaria disease cycle. This gene was down-regulated during early malaria and mildly up-regulated during recovery period.

**Table 4:** TH2 related gene unchanged after malaria

**U**	**E**	**E/U**	**A**	**A/U**	**R**	**R/U**	**Gene**	**TH2Immune response**
1.09	1.06	0.972	0.97	0.889	1.02	0.935	IL4	IL4 CD4 Tcell
1.07	1.08	1.009	0.97	0.906	1	0.934	IL4R	
1.02	1.08	1.058	0.99	0.970	0.97	0.950	IL5	
1.06	1	0.943	0.98	0.924	0.99	0.933	IL5R	
1.08	1.1	1.018	0.95	0.879	0.92	0.851	IL9	
1.08	1.08	1	0.99	0.916	0.95	0.879	IL9R	
0.94	0.99	1.053	1.15	1.223	1.09	1.159	IL10	
0.93	0.85	0.913	1.08	1.161	1.17	1.258	IL10RA	
1.09	1.05	0.963	0.95	0.871	0.92	0.844	IL13	
0.98	1.14	1.163	1.23	1.255	0.99	1.010	IL13RA1	
1.13	1.01	0.893	1.01	0.893	1.01	0.893	IL13RA2	
1.1	1.13	1.027	0.91	0.827	0.96	0.872	CCR3	
1.08	0.96	0.888	0.98	0.907	1.01	0.935	CCR4	
1.11	1.2	1.081	0.86	0.774	0.95	0.855	CCR8	
1.08	1.12	1.037	0.89	0.824	0.94	0.870	CCL17	
0.99	1.1	1.111	0.91	0.919	0.95	0.959	CCL22	
1.09	1.04	0.954	0.92	0.844	0.93	0.853	CRTH2	
1.05	0.94	0.895	1.08	1.028	0.98	0.933	CD30	
0.92	0.98	1.065	1.13	1.228	1.17	1.271	OX40	
1.32	1.31	0.992	0.93	0.704	0.89	0.674	OX40L	
1.01	0.88	0.871	1.01	1	0.99	0.980	STAT6	
1	0.92	0.92	1.18	1.18	1.16	1.16	GATA3	
0.98	1	1.020	0.96	0.979	1	1.020	NFATc	
0.92	0.91	0.989	1.27	1.380	1.31	1.423	Gfi-1	
0.96	1.09	1.135	1.1	1.145	1.07	1.114	SPHK1	
1.05	1.02	0.971	0.97	0.923	1	0.952	SH2D1A	
1.12	1.08	0.964	0.87	0.776	0.85	0.758	ST2L	
1.11	1.06	0.954	0.94	0.846	0.98	0.882	A3AR	Mast cell
1.1	1.13	1.027	0.91	0.827	0.96	0.872	CCR3	
1.03	1.22	1.184	0.89	0.864	0.99	0.961	FCER2	
1.14	0.63	0.552	1.17	1.026	1.72	*1.508*	FCER1A	
1.09	1.13	1.036	0.93	0.853	1.01	0.926	LTC4S	
0.95	0.96	1.010	1.11	1.168	1.05	1.105	MGST2	
0.89	0.93	1.044	1.19	1.337	1.13	1.269	MGST3	
0.92	0.92	1	0.84	0.913	0.85	0.923	CysLTR1	
1.07	1.04	0.971	0.94	0.878	0.94	0.878	CysLTR2	
1.02	1.08	1.058	0.99	0.970	0.97	0.950	IL5	Eosinophil
1.11	1.01	0.909	0.97	0.873	1.02	0.918	Eotaxin1	
1.18	1.17	0.991	0.88	0.745	0.89	0.754	Eotaxin2	

Italic means 1.5-2-fold change.

Plane means no change.

Underline means down-regulation.

### Gene-to-gene relationship

**Figure 2:**
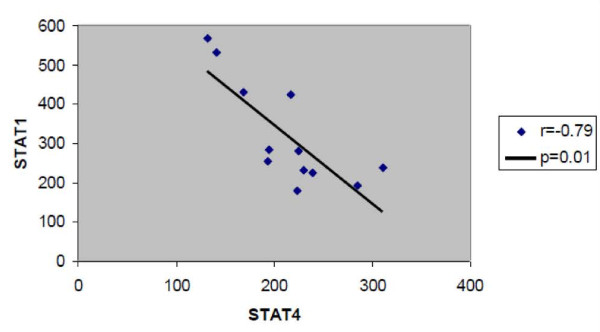
**Gene-to-gene correlation.** This figure is to compare STAT1 and STAT4 gene correlation in acute stage of malarial infection. These twelve samples are from patients are Cameroon acute malaria patients. There is negative correlation between STAT1 and STAT4, Pearson’s correlation coefficient (r) and its P value (p) were calculated for the graph.

This study performed Pearson’s correlation to analyse gene-to-gene relationship after malaria infection and found a number of relationships in the study subjects that were consistent with observations by other investigators in previous studies. This study observed a strong negative Pearson’s correlation between the expression levels of STAT1 and STAT4 in early malaria infection and acute febrile malaria (Figure 2). There are positive Pearson’s correlations between the expression level of STAT1 and Fc receptors (ADCC-related genes), but negative correlations between STAT4 and Fc receptors. These observations suggested STAT1 up-regulated Fc receptors. There is negative correlation between STAT1 and STAT4. Although some previous studies found that STAT4, IL-12R, and Fc receptors are co-expressed, STAT4 is not correlated or even negatively correlated with these up-regulated Fc receptors in malarial infection. Fc receptors suppressed STAT4 expression or STAT4 suppressed Fc receptors (Table 5). Rank correlation has also been done, but there were no significant results. Rank correlation is stricter than Pearson’s correlation. If the data are normally distributed, Pearson’s correlation analysis is more suitable than Rank correlation analysis.

**Table 5:** Correlation between Fc receptor/complement receptor and STAT1/STAT4 in acute febrile malaria

**Gene**	**STAT1**	**STAT4**
FCER1G	0.9 (0.001)	-0.71 (0.01)
FCGR1A	0.88 (0.001)	-0.74 (0.01)
FCGR2A	0.79 (0.01)	-0.79 (0.01)
FCGR2B	0.74 (0.01)	-0.77 (0.01)
Fas	0.71 (0.01)	-0.64 (0.05)
C3AR	0.86 (0.001)	-0.73 (0.01)
DAF	0.64 (0.05)	-0.66 (0.02)
C1QA	0.71 (0.01)	-0.64 (0.05)
Properdin	0.82 (0.01)	-0.68 (0.02)

### Immunological pathways

**Figure 3:**
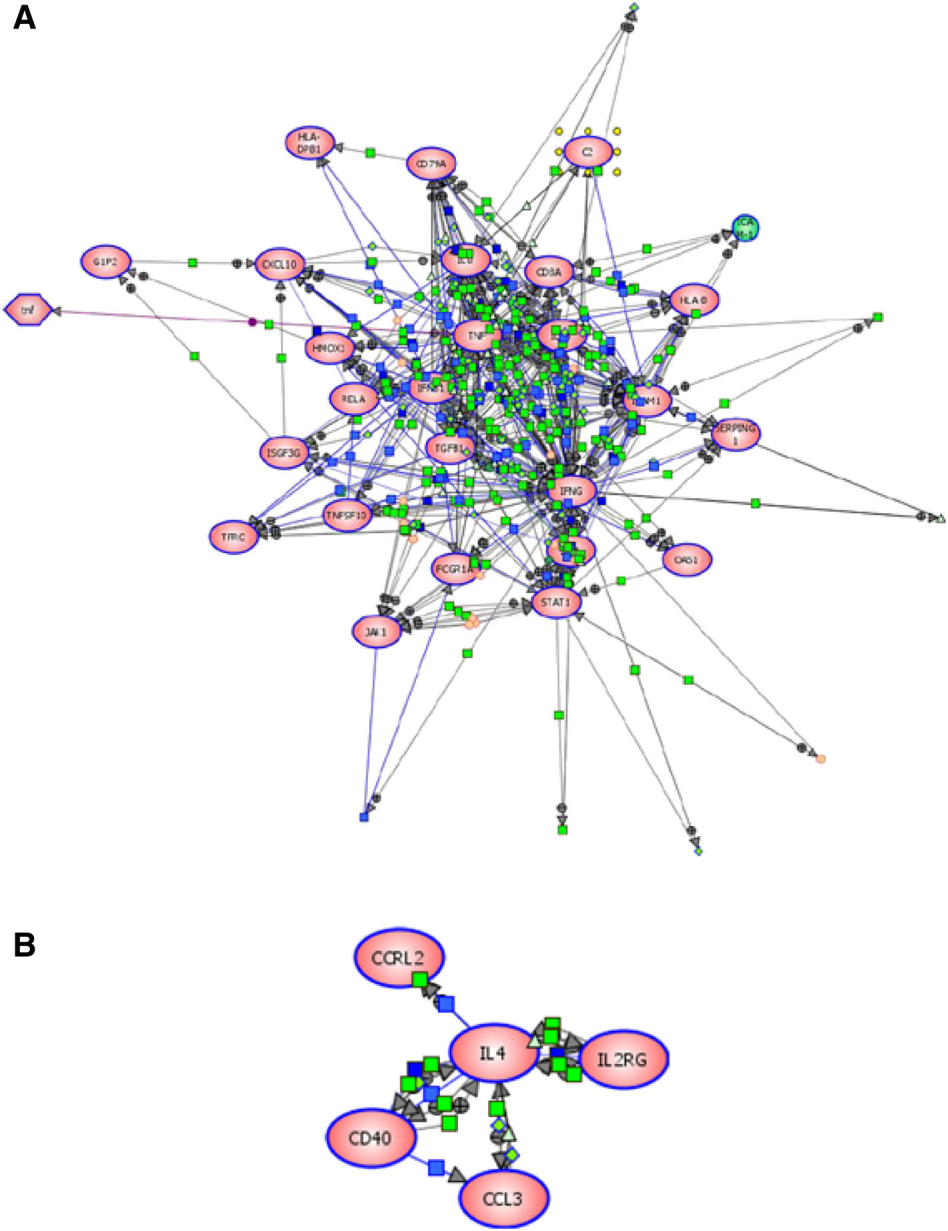
**IFNα/β and IL-4 centred pathway analysis. A** IFN-αβ centred pathway analysis. Network analysis was performed by using IFN-α and IFN-β as the central nodes. This study used software to select IFN-α or IFN-β related genes from the gene list. 24 IFN-α or IFN-β related genes were selected. Red entities mean proteins. Green entities mean small molecules including phospholipids and drugs. Hexon means groups of family proteins. **A**---**B** means **A** binds to **B** to form a complex. **A**- → **B** means **A** binds **B**’s promoter region to up-regulate **B**. **A**-- + - > **B** means **A** induces **B** release. **A**---|**B** means **A** causes **B**’s degradation or inhibition. Little square in the middle of the line means there is a third molecule which regulates the conversion from **A** to **B**. **B** IL-4 centred pathway analysis. Network analysis was performed by using IL-4 as the central nodes. This study used software to select IL-4 related genes from the gene list. Only four IL-4 related genes were selected.

**Figure 4:**
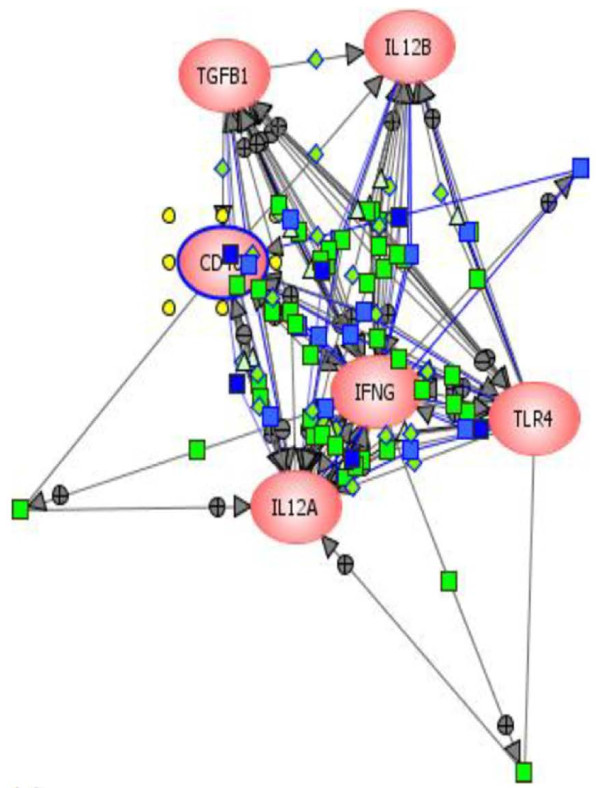
**IL-12 centred network analysis.** IL-12 centred pathway analysis. Network analysis was performed by using IL-12 as the central nodes. This study used software to select IL-12 related genes from the gene list. Only four IL-12A/IL12B related genes were selected.

**Figure 5:**
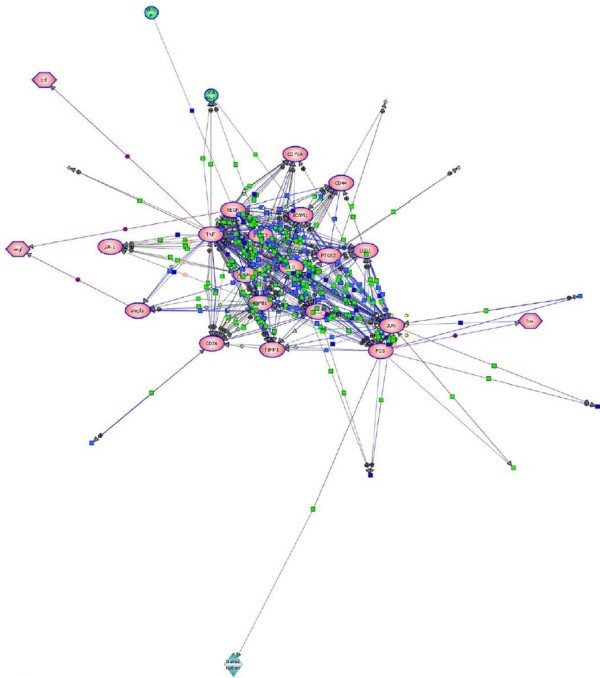
**TGF-β centred network analysis.** TGF-β/oncostatin M centred pathway analysis. Network analysis was performed by using TGF-β/oncostatin M as the central nodes. This study used software to select TGF-β/oncostatin M related genes from the gene list. 22 TGF-β/oncostatin M related genes were selected. Red entities mean s proteins.

**Figure 6:**
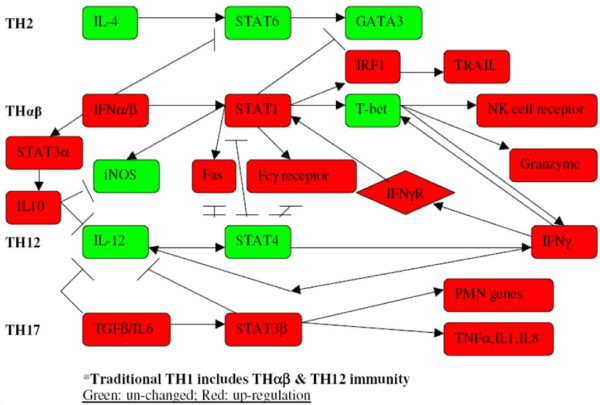
**Proposed model for the four types of host immune response.** Green means unchanged and red means up-regulated genes after malaria infection. TH2 immune response is induced by STAT6 activated by IL-4. THαβ immune response is induced by STAT1 & STAT3α activated by IFN-αβ. TH1 immune response is induced by STAT4 activated by IL-12. TH17 immune response is induced by STAT3β activated by TGF-β/IL-6 to induce downstream PMN related genes and pro-inflammatory cytokines (TNFα, IL1, IL8). In THαβ immune response, IFN-α/β can suppress STAT6. IFN-α/β also suppress iNOS and IL-12 via IL-10 dependent mechanism. Up-regulated STAT1 can suppress GATA3 and STAT4. STAT1 can up-regulate IRF1, T-bet, IgG Fc receptors, Fas, C3a receptor. IRF1 induced TRAIL, T-bet induced NK cell killer receptors, and Fas can enhance NK cell cytotoxicity. Up-regulated IgG Fc receptors can mediate ADCC. In T cell, T-bet can up-regulate IFN-γ. Up-regulated IFN-γ can activate STAT1 signalling via IFN-γ receptor. However, the IFN-γ receptor is suppressed during lymphocyte maturation. Thus, THαβ immune response can be separated from TH1 immune response.

**Figure 7:**
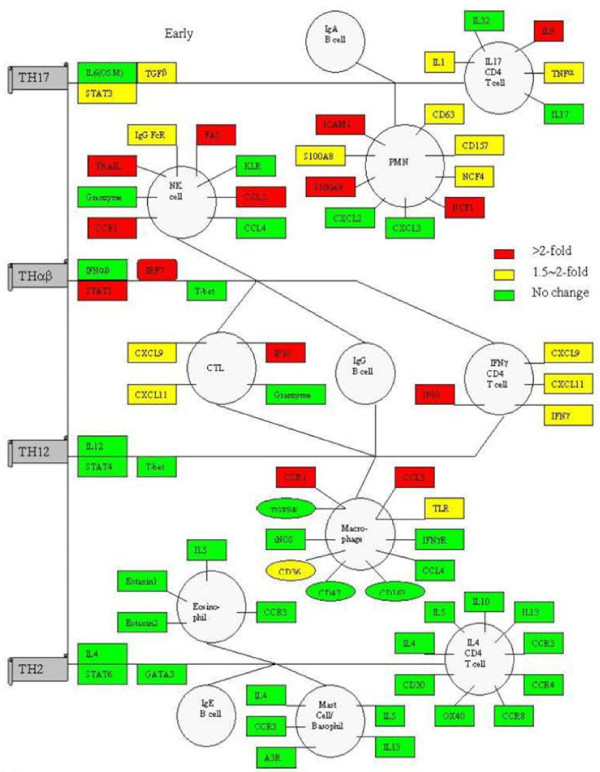
**Gene expression during early malarial infection, The meaning of colours is stated as Figure**1**.** This figure summarized the expressed genes of the four immunological pathways in early malarial infection.

**Figure 8:**
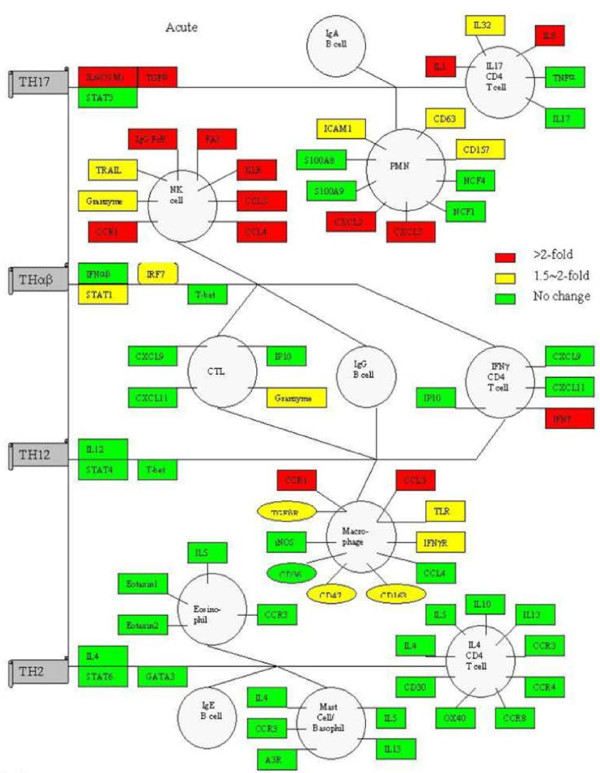
**Gene expression during acute febrile malarial infection, The meaning of colours is stated as Figure**1**.** This figure summarized the expressed genes of the four immunological pathways in acute febrile malarial infection.

**Figure 9:**
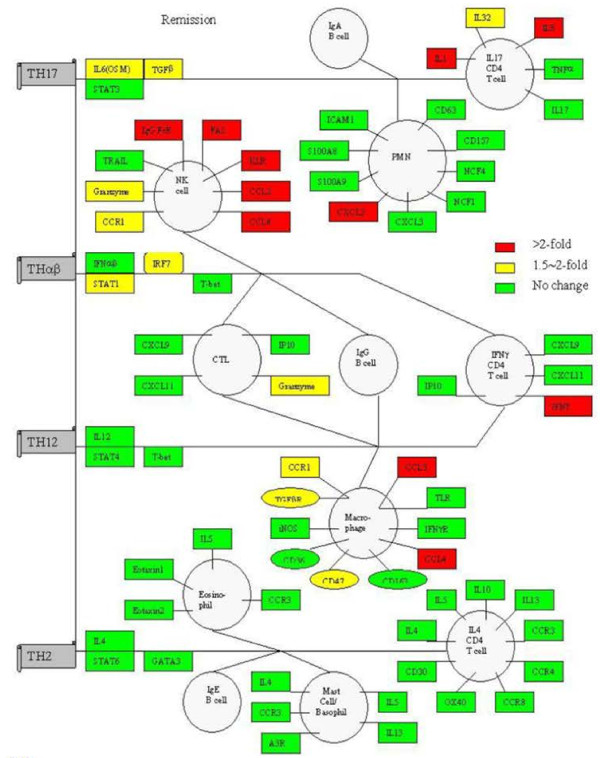
**Gene expression during the recovery period.** The meaning of colours is stated as Figure 6. This figure summarized the expressed genes of the four immunological pathways in recovery period after malarial infection.

Pathway analysis was conducted to explore immunological genetic circuitry during malaria infection. This study selected IL-4, IL-12, TGFB1/IL-6, IL-17, IFN-α/β as the central gene markers to explore possible networking among the 2,894 genes under analysis in this study. This study was able to identify only a few up-regulated genes in this cluster that centred on IL-4 (Figure 3) or IL-12 (Figure 4). In contrast, gene networks centred on TGFB1/IL6, IL-17 (Figure 5), or IFN-αβ (Figure 3) were more dramatically altered during the malaria disease cycle. These results further support a model for malaria immune response in which IL-17/IFN-alpha/beta plays a more central regulatory role than IL-4 and IL-12. Figure 6 shows a sketch of the major pathways of host immune response that are felt to play a role in recovery from malaria based on the observations made in this study, as well as those made by other investigators in previous research. The changes in their expression levels during the early, acute and recovery stages of *P. falciparum* infection are summarized in Figure 1. As shown in Figures 1, 7, 8 and 9, the expression of genes related to TH2 immune response were unchanged following malaria infection, with most changes occurring in genes related to the TH17 and THαβ driven pathway of anti-malaria immune response.

### Validation by plasma and PBMC protein expression

In the previous analysis, this study found out the concordance between transcription of genes and translation of proteins in malarial infection. This study demonstrated the similar up-regulation of ICAM1 level after malarial infection between microarray RNA result and surface protein expression detected by flow cytometry. CXCL10, CXCL9, and CCL2 chemokine expressions are up-regulated in this microarray finding. The above chemokine proteins are also identified in plasma samples after malarial infection. 4These results show that this microarray analysis is validated as shown in Figure 4 in [7].

## Discussion

Host immune response can be categorized into TH1, TH2, and TH17 immune response pathways. TH1 immune responses (Type 1 immune response) are IFN-γ and IgG-mediated and they play an important role in host responses to viruses and intracellular bacteria/protozoa. TH2 immune responses are IL-4 and IgE-mediated immune response are important to host responses to helminths; TH17, initiated by TGF-β, IL-6 and IL-17 are important in host responses to extracellular bacterial pathogens [9-11]. In this research, these data suggest that there should be a new immunological pathway derived from the original TH1 immune response: THαβ. THαβ is named after IFNα/β. In this model, THαβ is triggered by interferon alpha/beta + IL-10 and TH1 is triggered by interleukin 12. Based on previous work by others, THαβ is important in host immune response against virus infection; TH1 is important in host immune response against intracellular bacteria/protozoa. Different types of host immune response have different effector cells. In TH2 immune response, the effector cells are mast cells, eosinophils, basophils, IgE/IgG4 secreting B cells, and IL-4 secreting CD4 T cells. In TH17 immune response, the effector cells are neutrophils, IgA/IgM/IgG2 secreting B cells, and IL-17 secreting CD4 T cells. In THαβ immune response, the effector cells are NK cells, IgG1 secreting B cells, CTL, and IL-10 (+++)/IFN-γ(+) secreting CD4 T cells. In TH1 immune response, the effector cells are macrophages, IgG3 secreting B cells, CTL, and IFN-γ(+++) secreting CD4 T cells. Similarly different types of host immune response use different STAT proteins to initiate specific immune reactions. IL-4 activates STAT6 in TH2 immune response. TGF-β and IL-6 activates STAT3β in TH17 immune response. Interferon alpha/beta and IL-10 activates STAT1 and STAT3α in THαβ immune response. IL-12 activates STAT4 in TH1 immune response [12]. TGF-β and IL-2 activate STAT5 in regulatory T cell (Treg) immune response. Based on these data, malaria infection appears to induce a mixed TH17 and THαβ immune response which best fits with a model in which both pathways are activated during the onset and recovery from malaria infection.

Others have shown that TGF-β with IL-6 can induce TH17 immune response [10,11]. TH17 lymphocytes also produce large amounts of IL-8, TNF-α and IL-1, which recruit and activate neutrophils. Due to’ previous analysis, toll-like receptor 1, 2, 4, 7, 8 are up-regulated after malarial infection. TLR1/2/4 mainly induce anti-extracellular bacteria TH17 immune response. TLR7/8 binding to single strand RNA mainly induce anti-virus THαβ immune response. In addition, this study found a lot of heat shock proteins are up-regulated after malarial infection. Heat shock proteins, mainly HSP60 or HSP70, can activate TLR2/4 for inducing TH17 immune response. In this study, TGF-β, IL-1, and IL-8 were strongly up-regulated in acute malaria (Table 3). Although IL-17 was not up-regulated, the IL-6-related cytokine family gene oncostatin M, was up-regulated in acute malaria infection. oncostatin M can up-regulate STAT3β, a key mediator in TH17 immune response [13]. Thus, it appears that TH17 immune response can be induced in malaria infection. Up-regulated TGF-β, IL-6, or TNF are associated with a higher frequency of malaria complications and an overall poor malaria prognosis. A previous study revealed that neutrophil-related genes were up-regulated in children after acute *P. falciparum* malaria infection [14]. They also reported up-regulation of IL-6 receptor, C3AR1, C5R1, FPRL1, PBEF, and IL-1β, which are important components of TH17 immune response. Neutrophilia has been observed in falciparum malaria, and elevated white blood cell counts are associated with severe malaria [15]. Gene network analysis of TGFB1/IL-6 or IL-17-related gene expression after malaria infection also suggested that malaria was TH17 dependent. Falciparum malaria often causes complications such as acute renal failure and acute respiratory distress syndrome (ARDS). Neutrophil over-activation in TH17 immune response is thought to play a major role in the pathogenesis of cerebral malaria, acute renal failure, and ARDS [16-18]. Thus, malaria-induced TH17 immune response could be related to both malaria-induced cerebral malaria, acute renal failure and ARDS.

Evidence for dividing the more traditional TH1 immune response pathway into two subtypes (THαβ and TH1 immune response) comes primarily from both mouse and human models of malaria immune response. The major difference separating TH1 immune response and THαβ immune response are the effector cells involved. In the mouse model, interferon alpha/beta suppresses macrophage proliferation and neutrophiles and NK cells play a more prominent role, in contrast, IL-12 enhances macrophage proliferation and human derived macrophages can inhibit NK cells [19,20]. In addition, interferon alpha/beta increases NK cell blastogenesis, so alpha/beta interferon enhances NK cell proliferation [21]. Thus, interferon alpha/beta and IL-12-mediated immunological events can be distinguished by the different effector cells they enhance, NK cells or macrophages [22].

Mouse models of anti-malaria immune response indicate that interferon gamma and TH1 immune response play central roles in the developmental process [2]. Although administration of IL-12 can provide 100% protection against malaria parasite challenge, IL-12 plays a limited role in natural immune response against malaria in mice [1]. Active immunosuppression is well documented during malaria infection in mice [3]. IL-12 expression is down-regulated in this model [23]. According to an IRF1 knock-out study, mice can initiate TH1 immune response by bypassing the need for IL-12 production through the use of an alternate interferon alpha/beta driven pathway [24]. Based on recent microarray studies in malaria-infected mice, there is no evidence of IL-12 up-regulation following infection. Instead, interferon alpha/beta and its related genes were found to be significantly up-regulated [25,26], thus providing further evidence at the gene level that interferon alpha/beta can substitute for IL-12 in the induction of malaria-specific interferon gamma TH1 type immune response after infection in rodents. However, the IFNαβ driven THαβ immune response only can produce mid-to-moderate IFNg, and IL-10, the main effector molecule in THαβ immune response, antagonizes the effect of IFNg upon macrophages. Because the ideal traditional TH1 immune response cannot be triggered in natural malarial infection in human to clear out the protozoa, malaria parasites can cause severe illness in human.

In human, there is also a substantial amount of evidence indicating that TH1 immune response can be induced by two pathways driven by αβ interferon or IL-12 [27,28]. Administration of IL-12 to human subjects suppresses NK cell proliferation [29]. Macrophage activation serves to inhibit NK cell function, while NK cell activation serves to inhibit macrophage proliferation [19,30]. Given these observations, it is reasonable to speculate that these two effector cells belong to different immunological pathways. In the present study, this study found that a number of genes involved in the NK cell proliferation were up-regulated over the course of the malaria disease cycle (Table 1). In addition, as in the mouse model, genes associated with macrophage proliferation were not up-regulated following infection with *P. falciparum*. Increased NK cell populations have also been reported previously in acute malaria infection [31,32], thus, it appears that the interferon alpha/beta-NK cell pathway of THαβ immune response is preferentially enhanced after *P. falciparum* infection, while the IL-12 driven pathway appears unaffected. Other THαβ-related cytokines also can cause NK cell activation or proliferation including IL-10 and IL-15, respectively [33].

In human malarial infection, it has been shown that serum IL-12 levels are inversely correlated with malaria parasitaemia [6]. The more severe the malaria is, the lower IL-12 expression levels become. The uptake of malarial pigment (haemozoin) can down-regulate IL-12 secretion in human monocytes [5]. Malarial PGE2-like molecules can also down-regulate IL-12 secretion [34]. Up-regulation of CD36 by malarial infection can inhibit dendritic cell maturation with decreasing IL-12/IL-10 ratio [4]. Although haemozoin, in certain studies, can activate TLR9, which is for traditional TH1 immune response, TLR9 was not detected in this study. In previous microarray analysis of human malarial infection, interferon inducible genes were up-regulated with unchanged IL-12 expression [14,35]. Other important components of THαβ immune response included up-regulation of HMOX1, FAS, BCL6, TNFRSF10A, and MIP1α were noted in Griffiths’ research [14], which studied the peripheral blood leukocytes from Kenyan children with acute falciparum malaria. In this microarray study, this study ound up-regulation of interferon alpha/beta, interferon gamma, NK cell activating receptors, Fas, TRAIL, Fc gamma receptors, ADCC-related genes, IL-15, granzymes, and TH1 related chemokines (IP10, CXCL9, CXCL11, CCL3, CCL4, CCR1). These observations are, in general, consistent with the development of THαβ immune response characterized by NK cell activation induced by alpha/beta interferon following *P. falciparum* infection. Genes associated with TH1 immune response driven by IL-12 and macrophage activation (iNOS up-regulation) were unchanged after malarial infection. Gene coding for a number of macrophage de-activation molecules were up-regulated, including TGFβ receptor, CD36, CD163-Haem oxygenase1, and CD47-SIRPα. These molecules can actively suppress macrophage activation. When given alone and prior to malaria infection, IL-12 can provide 100% protection against experimental *Plasmodium* infection through the activation of macrophages, which help to control the liver stage of infection [1]. However, based on the data collected during the course of the current study, it also appears that infected individuals defend malaria through an alternate THαβ immune response, involving alpha/beta interferon and the activation of NK cells [36]. Interferon gamma plays a central role in immune response against malaria. However, the natural host immune response is suboptimal because IFN-γ is not the main effector in THαβ immune response. The major effector cells of THαβ immune response are NK cells, and the major effector cells of traditional TH1 immune response are macrophages.

Although IL-10, a strong macrophage de-activator, was not up-regulated in this study, many downstream genes of IL-10 were up-regulated including haem oxygenase 1, and it suggests that IL-10 is actually up-regulated after malaria infection. In an ideal situation, TH1 immune response is basically immune response against intracellular bacteria/protozoa; THαβ immune response is basically immune response against viruses. Although, it appears that macrophages may not be activated in natural immune response against malaria, interferon gamma(+) and IL-10(+++) secreting CD4 T cells, cytotoxic CD8 T cells, IgG secreting B cells, and NK cells are up-regulated by interferon alpha/beta in malarial infection. In a clinical study in Gabon, interferon-γ and IL-10 secreting CD4 T cells were increasing and correspondent to the recovery of *P. falciparum* infection [37]. Pure TH1 interferon-γ producing T cells and pure TH2 IL-4 producing T cells were not changed during convalescence after malaria infection. Thus observation is consistent with a model in which interferon-γ and IL-10 secreting T cells play important roles in host immune response against malaria.

## Conclusions

It appears that the human immune response to *P. falciparum* is characterized by a suboptimal THα/β and TH17 bias, which predominates over the more ideal and effective traditional TH1 responses driven by IL-12. Since treatment for malarial infection faces some difficulty, such as drug resistance, this study strongly encourage the use of IFNγ (FDA approval drug) or IL-12 for malarial infection treatment to achieve the optimal host immune response to kill the intracellular protozoa.

## Competing interests

The author has declared that he has no competing interests.

## Author’s contributions

W-CH, is responsible for the conception and design of this study, for data acquirement and analysis, for manuscript preparation, and for final approval and submission.
